# Assessment of SARS-CoV-2 vaccination status in SARS-COV-2 infected patients admitted in Dr Ruth K.M. Pfau, Civil Hospital Karachi

**DOI:** 10.12669/pjms.38.8.5733

**Published:** 2022

**Authors:** Tazeen Rasheed, Faiza Sadaqat Ali, Bader Faiyaz Zuberi, Rabiah Sadaf

**Affiliations:** 1 Tazeen Rasheed, FCPS. Associate Professor, Department of Medicine/Gastroenterology, Dow Medical College, Dow University of Health Sciences, Karachi, Pakistan; 2 Faiza Sadaqat Ali, FCPS. Senior Registrar, Department of Medicine/Gastroenterology, Dow Medical College, Dow University of Health Sciences, Karachi, Pakistan; 3 Bader Faiyaz Zuberi, FCPS. Meritorious Professor, Department of Medicine/Gastroenterology, Dow Medical College, Dow University of Health Sciences, Karachi, Pakistan; 4 Rabiah Sadaf, FCPS. Consultant Physician, Dr Ruth K.M. Pfau, Civil Hospital Karachi, Pakistan

**Keywords:** SARS-CoV-2, SARS-CoV-2 Vaccine, Mortality, Odds Ratio

## Abstract

**Objectives:**

To determine the frequency of vaccination status in patients with SARS-CoV-2 infection

**Methods:**

This case-control study was conducted at Dr Ruth KM Pfau Civil Hospital Karachi, Pakistan between September 2021 to October 2021. All patients who had positive PCR on nasopharyngeal swab for SARS-CoV-2 infection were included. Information regarding vaccination status and brand of vaccination administered and duration between the last dose of vaccine and positive PCR was noted. The disease status of patients was classified on admission into severe and non-severe disease.

**Results:**

Study included 143 patients, out of which 58 (40.6%) were males and 85 (59.4%) were females. Majority of our patients (78.3%) were unvaccinated. Frequency of Severe SARS-CoV-2 Infection in fully vaccinated patients was less than in unvaccinated patients. The odds of developing severe COVID infection in unvaccinated patients versus vaccinated was 8.55 times higher (OR = 6.23, 95% CI 2.58-28.35). Proportion of vaccinated females was less as compared to males. Significant differences were found in severity between hypertension (p<.001), diabetes (<.001) and age (p<.001).

**Conclusion:**

The frequency of SARS-CoV-2 infection was greater in unvaccinated patients. The odds of developing severe COVID infection in unvaccinated patients versus vaccinated was 8.55 times higher.

## INTRODUCTION

In November 2019, several cases of pneumonia of unknown etiology were reported in Wuhan, China. The causative organism of this pneumonia was later on identified as a beta coronavirus that was closely related to SARS virus first identified in 2003, hence this virus was termed SARS-CoV-2. SARS-CoV-2 is thought to have likely originated in bats but it might have replicated in an intermediate host.^1^

Coronavirus disease 2019 (COVID-19), is a highly contagious infectious disease caused by severe acute respiratory syndrome coronavirus 2 (SARS-CoV-2). This ongoing global pandemic has inflicted tens of millions of people worldwide with the numbers increasing daily.^2^ According to data by World Health Organization (WHO), as of November 8^th^ 2021, the count of confirmed COVID-19 cases was 249,507,923 with 5,044,654 deaths.^3^ This pandemic continues to progress worldwide however, many details about the disease dynamics remain obscure.^4^

Since its breakout there has been tremendous progress with the development, authorization and deployment of vaccines and antibody therapies. The emergence of different viral variants, particularly in the ‘*S* gene’, threatens the efficacy of vaccines.^5^ However, recently there have been cases reported of breakthrough infections among recipients of COVID-19 vaccines. A vaccine breakthrough infection is defined as the “Detection of SARS-CoV-2 RNA or antigen in a respiratory specimen collected from a person ≥14 days after receipt of all recommended doses of an FDA-authorized COVID-19 vaccine”.^6^ Recent studies of fully vaccinated individuals have shown that it does not result in complete prevention of SARS-CoV-19 infection but definitely marked reduction in transmission.^7^

In a Mini-review discussing the reliability and efficacy of different COVID vaccines, 19 studies were reviewed, and it was concluded that the efficacy of different vaccines is Pfizer–BioNTech is ~95%, Moderna is ~94%, Sputnik V ~92% and Oxford-AstraZeneca is ~81%. In the same review other vaccines Convidicea, Johnson & Johnson, Sinopharm, COVAXIN and Sinovac were discussed based on their immunogenicity, and safety.^8^ Vaccines produced by China which are inactivated whole virus vaccines, such as Sinovac Biotech (Sinovac, Beijing, China) and Sinopharm (Sinopharm, Beijing, China) are approved for use in China and some other countries. The Sinopharm vaccine trial has shown its efficacy at 86% and is being used in Bahrain, United Arab Emirates and Pakistan. Mixed reports were obtained for Sinovac’s vaccine termed CoronaVac, ranging from 50.4-78.0% efficacy in a Brazilian trial, 91.25% in Turkey and 65.3% in Indonesia.^9^ Single dose CanSino (adenovector) vaccine showed seroconversion rates of 96-97% & 88-90% for T cell responses.^10^

In Pakistan, Sinopharm, Sinovac, Moderna, Pfizer, AstraZeneca and single dose CanSino vaccines are being administered. Vaccination rate has been slow in Pakistan, at the time of writing this report only 6.8 million people in Pakistan had been vaccinated.^11^ Since there are very few studies showing the efficacy of these vaccines, therefore we conducted this study to determine frequency of vaccination in patients admitted with SARS-CoV-2 infections.

### Operational Definitions:

***Vaccine breakthrough infection:*** “Detection of SARS-CoV-2 RNA or antigen in a respiratory specimen collected from a person ≥14 days after receipt of all recommended doses of an FDA-authorized COVID-19 vaccine”.^6^

***Fully vaccinated:*** “Patients who received two doses of a two-dose vaccine series and ≥ 14 days had elapsed since the second dose OR who received single dose of CanSino vaccine and > 14 days had elapsed.^12^

***Partially Vaccinated:*** “Subjects who received one dose of a two-dose series”.^12^

***Severe SARS-CoV-2 Infection:*** “Oxygen saturation of <94 %or respiratory rate of over 25 breaths/minute”.^13^

***Non-severe SARS-CoV-2 Infection:*** “Oxygen saturation of 94% or greater and respiratory rate of less than 25 breaths/minute”.^13^

## METHODS

This Case-control Study was conducted at Dr Ruth KM Pfau, Civil Hospital Karachi between 15^th^ September, 2021 to 14^th^ October, 2021. Patients satisfying inclusion/ exclusion criteria were included afterwritten informed consent. Approval (IRB-2221/DUHS/Approval/2021/507-dated 14 -9-2021) was taken from Institutional Review Board of Dow University of Health Sciences. A total of 143 patients were included. Non- probability consecutive sampling was used for selection of patients.

### Inclusion criteria:

All patients who had positive PCR on nasopharyngeal swab for SARS-CoV-2 infection, admitted in COVID-19 Treatment Facility of Dr Ruth KM Pfau, Civil Hospital Karachi were included.

### Exclusion criteria:

Patients on immunosuppressive drugs and autoimmune diseases were excluded.

### Sample Size:

Using the reported 10% symptomatic infections requiring hospitalization in vaccinated people,^14^ power of 90% and Alpha of 0.05, the sample size was calculated as 12. Thus with 10% reported frequency a minimum of 120 patients were included to achieve the sample size of 12.

### Sampling Technique:

Non-probability consecutive sampling.

### Data Collection:

All admitted patients meeting inclusion criteria were included after informed consent. Demographic data of age and gender concurrent diseases like diabetes, hypertension was collected. Information regarding vaccination status and brand of vaccination administered and duration between the last dose of vaccine and positive PCR will be noted. SARS-CoV-2 vaccination status was classified into three categories as defined in the operational definitions as fully vaccinated, partially vaccinated and unvaccinated based on the vaccination status at the time a positive PCR test on nasopharyngeal swab.^12^ The disease status of patients was classified on admission into severe and non-severe disease as given in operational definitions.

### Data Analysis:

Data was analyzed using SPSS version 26. Frequencies of gender, diabetes, hypertension, vaccination status and if vaccination done its category and disease severity status was reported. Age, time lapse duration from vaccination till infection was reported in means ±SD. Cross tabulation of vaccination status with gender, diabetes, hypertension and disease severity was done and significance tested by χ^2^ test. Significance level was set at ≤0.05. Odds Ratios were calculated for Severe COVID infections for Diabetes, Hypertension and Vaccination Status.

## RESULTS

In this study one hundred and forty-three patients admitted in Covid-19 Treatment Facility of Dr. Ruth K.M. Pfau, Civil Hospital Karachi were included. Mean ±SD of age of the patients was 52.88 ±16.29 years. Out of the 143 patients 58 (40.6%) were males and 85 (59.4%) were females. Mean age of males was 53.62 ±16.04 years while that of females was 52.38 ±16.54 years. The difference in age among genders was not statistically significant (*p* = 0.656; df 141; 95% CI -6.75 to 4.25).

Out of our total of 143 patients 19 (13.3%) patients were fully vaccinated, 12 (8.4%) patients were partially vaccinated &112 (78.3%) were unvaccinated. Out of 112 unvaccinated patients 70(62.5%) were females and 42(37.5%) were males. Among the vaccinated patients (fully vaccinated, partially vaccinated) 20 (14.0%) patients received Sinopharm, four (2.8%) received Sinovac, four (2.8%) received CanSino, one (0.7%) received Moderna, two (1.4%) received Pfizer.

As shown in Table-I among the unvaccinated females 14 (20%) developed non severe SARS-CoV-2 infection and 56 (80%) developed severe SARS-CoV-2 infections. In fully vaccinated females, six (75%) developed non severe SARS-CoV-2 infection and two (25%) developed severe SARS-CoV-2 infection whereas, Among the fully vaccinated males, seven (63.6%) developed non-severe SARS-CoV-2 infection and four (36.4%) developed severe SARS-CoV-2 infections whereas, among the unvaccinated males 17 (40.5%) developed non- severe SARS-CoV-2 infection and 25 (59.5%) developed severe SARS-CoV-2 infection.

**Table-I:** Frequencies of SARS CoV2 infection according to Vaccination Status and Gender.

		Non-Severe SARS CoV2 infection		Severe SARS CoV2 infection		Row Total	p-value*

		n	%	n	%	N	
Unvaccinated	Female	14	20.0%	56	80.0%	70	0.019
	Male	17	40.5%	25	59.5%	42	
Fully Vaccinated	Female	6	75.0%	2	25.0%	8	0.596
	Male	7	63.6%	4	36.4%	11	
Partially Vaccinated	Female	0	0.0%	7	100.0%	7	0.170^†^
	Male	1	20.0%	4	80.0%	5	
Column Total		45	31.5%	98	68.5%	143	

* Significance level ≤.05,

† Likelihood Ratio.

Out of the total of 143 patients 45 (31.5%) of the patients had non-severe SARS-CoV-2 Infection whereas 98 (68.5%) of the patients had severe SARS-CoV-2 infection. Sixty-five (66.3%) females had severe SARS-CoV-2 infection as opposed to 33 (33.7%) males had severe SARS-CoV-2 infection. The difference in the severity of infection among genders was statistically significant (*p*= 0.013). Females had 2.462 times increased odds of developing severe SARS-CoV-2 infection. (OR = 2.462, 95% CI 1.20-5.07).

The mean age of patients having non-severe SARS-CoV-2 infection was 43.98+17.44 whereas the mean age of patients having severe SARS-CoV-2 infection was 56.97 +14.04. There is significant difference in the disease severity with regards to increase in age (p <0.001; df 141; 95% CI 7.08 to 18.89).

Out of the 143 patients 79 (55.2%) were hypertensive and 64 (44.8%) were non-hypertensive. Among the hypertensive patients 67(84.8%) had severe SARS-CoV-2 infection whereas 12 (15.2%) patients had non-severe SARS-CoV-2 Infection. The difference between the severity of infection between hypertensive and non- hypertensive patients was statistically significant [χ^2^ (df=1, N=143) = 21.69; *p<0.001*]. Hypertensive patients had 5.94 times increased odds to have severe SARS-CoV-2 infection as compared to non-hypertensive patients (OR = 5.944, 95% CI 2.71-13.04). Table-II

**Table-II:** Odds of SARS CoV-2 Severe Infection with Vaccination Status, Diabetes Mellitus, Hypertension& Gender.

		Disease Severity				*p* value*	Odds Ratio

		Severe		Non-Severe			

		n	%	n	%		
Vaccination Status	Unvaccinated	92	74.2%	32	25.8%	<.001	6.229^†^
	Vaccinated	6	31.6%	13	68.4%		
Diabetes Mellitus	Diabetic	52	89.7%	6	10.3%	<.001	7.348^‡^
	Non-Diabetic	46	54.1%	39	45.9%		
Hypertension	Hypertensive	67	84.8%	12	15.2%	<.001	5.944^§^
	Non-hypertensive	31	48.4%	33	51.6%		
Gender	Female	65	76.5%	20	23.5%	.013	2.462**
	Male	33	56.9%	25	43.1%		

* Significance Level ≤.05,

† Odds of Vaccination Status (Unvaccinated/Vaccinated) for Severe Disease,

‡ Odds of Diabetic Status (Diabetes/Non-diabetes) for Severe Disease,

§ Odds of Hypertension Status (Hypertension/Non-hypertension) for Severe Disease,

** Odds of Gender (Female/Male) for Severe Disease.

Among the diabetic patients 51(89.5%) had severe SARS-CoV-2 infection and six (10.5%) patients had non-severe SARS-CoV-2 Infection. The difference in the severity of infection between diabetic and non-diabetic patients was statistically significant [χ^2^ (df=1, N=143) = 20.189; *p<0.001*]. The odds of diabetic patients were 7.21 times higher to develop severe SARS-CoV-2 infection as compared to non-diabetics (OR = 7.348, 95% CI 2.85-18.94). Table-II.

Frequency of Severe SARS CoV2 Infection in Fully Vaccinated Patients was six (6.1%), 81 (82.7%), in Unvaccinated patients, 11 (11.2%) in Partially Vaccinated patients. The difference in the severity of infection between the vaccinated and unvaccinated patients was significant (*p*<.001) Table-II. For the calculation of Odds Ratio, the data of partially vaccinated and unvaccinated was merged with unvaccinated category. The odds of developing severe covid infection in unvaccinated patients versus vaccinated was 6.23 times higher (OR = 6.229, 95% CI 2.18-17.76). Table-II

Evaluation of disease severity with brand of vaccination does not show any significant difference with p=0.208. Details are given in Table III.

**Table-III:** Disease severity according to the brand of vaccination.

		Disease Severity		Total

		Severe	Non severe	
Vaccine Brand	None	81	31	112
	Sinopharm	10	10	20
	Sinovac	2	2	4
	CanSino	2	2	4
	Moderna	1	0	1
	Pfizer	2	0	2

Total		98	45	143

## DISCUSSION

Since vaccines are among the most effective public health measures against infectious disease therefore, vaccines are thought to be the best possible solution for controlling this ongoing pandemic. Generally, vaccine development progresses through several steps, each step occurs sequentially, and each usually takes several years for completion. However, covid-19 vaccine development has accelerated at an unprecedented pace.

Till now several effective vaccines against COVID-19 have been developed and approved. Mass vaccination campaigns to prevent COVID-19 are now occurring in many countries.^16^ Preliminary results of the effectiveness of other COVID-19 vaccines across different populations have been published, including studies at the national level in Israel^17^ and Scotland^16^ and studies involving essential frontline workers at specific locations in the United States.^18^

On December 8^th^ 2020, the United Kingdom was the first country to start a COVID-19 vaccination program after emergency use authorization of the BNT162b2 messenger RNA (mRNA) vaccine (Pfizer–BioNTech) by the United Kingdom’s Medicines and Healthcare Products Regulatory Agency.^19^ As of November 4^th^ 2021, a total of 7,027,377,238 vaccine doses have been administered worldwide.^20^

In this study we aimed to determine the frequency of vaccination in patients admitted with SARS-CoV-2 infections. One hundred and forty-three patients having SARS-CoV-2 infection were included in this study. In our study most patients were unvaccinated. Our results showed that the frequency of severe SARS-CoV-2 infections in unvaccinated patients was greater than that in vaccinated patients. These results were similar to a study done by Griffin JB HM et al. which included 43,127 patients having SARS-CoV-2 infections. Their data indicates that vaccines were protective against SARS-CoV-2 infection and severe COVID-19 infection. The results revealed that majority of patients having SARS-CoV-2 infection were unvaccinated and much lower percentage of fully vaccinated patients were hospitalized or admitted to intensive care unit (ICU).^21^

An increase in COVID-19 disease severity with increased patient age has been widely noted. An increased age-related risk of COVID-19 disease severity, admission to ICU, and death has been reported in several studies.^22^,^23^ In our study the mean age of patients having severe SARS-CoV-2 infection was higher than patients having non-severe SARS-CoV-2 infection.

Our study showed that hypertensive patients had 5.94 times more odds to develop severe SARS-CoV-2 infection as compared to non-hypertensive patients (OR = 5.94, 95% CI 2.71-13.04). Zhang J et al. also reported that severity of SARS-CoV-2 infection in hypertensive patients was higher than in non-hypertensive patients and hypertensive patients carried a nearly 3.48-fold higher risk of dying from COVID-19.^24^We in our study did not assessed difference in mortality between hypertensive and non-hypertensive patients but odds of developing severe disease was higher in hypertensive patients.

We also documented that odd of diabetic patients having severe SARS-CoV-2 infection as compared to non-diabetic patients was 7.35 times higher (OR = 7.35, 95% CI 2.85- 18.94). Our results were similar to a research article published in Diabetes, Obesity and Metabolism which showed that diabetes mellitus is associated with a higher risk of severity and fatality of COVID-19.^25^

In our study more females had severe SARS-CoV-2 infection as compared to males. Our results are contradictory to those by Jin JN et al. which showed that men with COVID-19 are more at risk for worse outcomes and death, independent of age. However, in this study only a case series of 43 patients with SARS-CoV-2 was included.^26^ Data from other studies also indicate that men suffer from more severe disease and have higher mortality than women.^27^,^28^ The reason cited for more severe infection in males is that men are more susceptible to pathogens while females mount a stronger antigenic response to infection, vaccines, and self-antigens at the cost of a higher prevalence of autoimmune disorders. In our study proportion of vaccinated females was less as compared to males. Among the unvaccinated study participants, the disease severity was more in females. However, in the fully vaccinated study participants the disease severity was more in males probably showing that after vaccination the females mounted a greater antigenic response to infection.

### Limitations of study:

The limitation of our study was that ours was a single center with a small sample size.

## CONCLUSIONS

The frequency of SARS-CoV-2 infection was greater in unvaccinated patients as compared to vaccinated patients. Unvaccinated females suffer from more severe disease as compared to males. The percentage of patients with severe disease increases with increase in age and presence of comorbids like diabetes and hypertension. Preventive measures along with mass vaccinations must be done in order to decrease hospitalizations and deaths from SARS-CoV-2 infections and thereby, control this pandemic.

### Authors’ Contribution:

**TR, BFZ, RS: C**onception and design, acquisition of data, analysis and interpretation of data.

**FSA, TR:** Drafting the article, revising it critically for important intellectual content.

**BFZ:** Final approval of the version to be published.

**TR, FSA:** Statistical Analysis.

All Authors Agree to be accountable for all aspects of the work in ensuring that questions related to the accuracy and integrity of any part of the work are appropriately investigated and resolved.
